# The Effect of Different Feeding Systems on Salivary Cortisol Levels during Gestation in Sows on Herd Level

**DOI:** 10.3390/ani11041074

**Published:** 2021-04-09

**Authors:** Ida Bahnsen, Kristina V. Riddersholm, Leonardo V. de Knegt, Thomas S. Bruun, Charlotte Amdi

**Affiliations:** 1 Department of Veterinary and Animal Sciences, Faculty of Health and Medical Sciences, University of Copenhagen, Grønnegårdsvej 2, 1870 Frederiksberg, Denmark; idabahnsen@yahoo.dk (I.B.); riddersholm95@hotmail.com (K.V.R.); lvdk@sund.ku.dk (L.V.d.K.); 2 SEGES Danish Pig Research Centre, Axeltorv 3, 1609 Copenhagen, Denmark; thsb@seges.dk

**Keywords:** feeding system, gestation, salivary cortisol, sows, stress

## Abstract

**Simple Summary:**

Physiological stress increases the activity of the hypothalamic-pituitary-adrenal (HPA) axis and the secretion of cortisol, which might cross the placenta and affect foetal development. Stress in sows can be affected by management factors such as enrichment, different feed systems of the housing accommodation, and is reflected in the salivary cortisol concentration. It is unclear how stressed the sow must be before there is an impact on foetal growth, but higher levels of cortisol might affect the maturity of piglets at birth as well as their birth weight. Therefore, it could be beneficial to accommodate gestating sows in the least stressful manner, not only for piglet performance but also for sow welfare. Cortisol concentration in sows seems to be influenced by a combination of parity and feed systems, but its connection to those factors, as well as to foetal development, warrants further investigation.

**Abstract:**

The aim of this study was to investigate herd cortisol levels as an indicator of stress during gestation in three different feeding systems. Twelve commercial Danish herds with 800 to 3050 sows were included, with either free-access feeding stall (Stall), floor feeding (Floor), or electronic sow feeding (ESF; *n* = 4 herds per system). Saliva samples were collected from 30 sows/herd in the gestation unit for cortisol analysis with an average of 67.2 gestation days for ESF, 72.4 days for Floor, and 68.6 days for Stall. Data on piglet birth weight (PBW) and the percentage of intrauterine growth restricted (IUGR) piglets from 452 litters (9652 piglets, 8677 liveborn) from all 12 herds were obtained on the saliva collection days. The cortisol levels in saliva increased throughout gestation (*p* < 0.01), and lower concentrations were observed among sows belonging to Stall (4.80 nmol/L), compared to Floor (7.03 nmol/L) and ESF (7.87 nmol/L), and that difference was significant as an independent effect in the case of ESF (*p* < 0.01). There was no difference between Floor and ESF or Stall and Floor (*p* > 0.05). An interaction was observed between parity and feeding system, with parities 4–5 in ESF herds having lower levels than other parities within the ESF system (*p* = 0.02).

## 1. Introduction

Piglet mortality is an ongoing concern of the Danish pig production, and piglets that have a low piglet birth weight (PBW), suffer from intrauterine growth restriction (IUGR) or are born of litters with a high within-litter variation of PBW (PBW_CV_) have an increased risk of dying before weaning [1,2,3]. It is therefore of interest to investigate factors that could potentially influence and result in a low PBW, a high PBW_CV_, or a high occurrence of IUGR piglets so that these parameters can be improved. Piglets suffering from IUGR have not received enough nutrients during development, and their brain is prioritised for the survival of the organism [4], giving them their characteristic head shape [2,5,6]. It is not known when during gestation this takes place although recent studies suggest differences in the development of porcine foetuses already at day 28 of gestation (Strathe et al., unpublished). 

Physiological stress increases the activity of the hypothalamic-pituitary-adrenal (HPA) axis and the secretion of glucocorticoid hormones, namely cortisol [7], which might cross the placenta and affect foetal development [8]. The exact mechanisms behind this are unknown, but it has been suggested that a higher transfer of cortisol across the placenta will limit foetal insulin-like growth-factor-1 (IGF-1) since cortisol is an inhibitor for IGF-1 and, thereby, affects foetal development [9]. Further, preterm growth-restricted babies display alterations in the growth hormone (GH)–IGF-1 axis (with increased GH and low IGF-1 concentrations) [10]. It is, therefore, possible that increased levels of cortisol could inhibit growth (measured by birth weight) and development (measured by IUGR). 

Several biological factors could potentially affect cortisol levels, and thereby growth and development of the foetus and ultimately sow performance. For example, Roelofs et al. [11] reported higher salivary cortisol concentrations for primiparous sows when compared with multiparous sows, and Strawford et al. [12] reported that concentrations are lowest in the intermediate sows (2nd to 3rd parity), compared to younger (1st parity) and older sows (≥4th parity). In addition, time of sampling during gestation as well as feeding system might influence salivary cortisol concentrations. Anil et al. [13] found that cortisol levels were higher at day 108 than at days 28, 56, and 84 of gestation in individually fed sows, whereas Holt et al. [14] found a decrease between days 40 and 80 of gestation. For sows housed in an Electronic sow feeding (ESF) system, saliva cortisol concentrations were higher at day 108 than at days 28 and 56, but not higher than at day 84 of gestation [13]. Moreover, it was reported that cortisol concentration measured by hair samples of sows in late gestation increases with litter size [11]. Thus, saliva cortisol concentrations during gestation might vary, depending on biological circumstances.

There was also evidence that feeding systems and housing could influence salivary cortisol levels and that this, in turn, might influence the piglet performance. Merlot et al. [15] found higher levels of cortisol in sows housed under barren systems (a conventional French system on slatted floors) compared to an enriched system with larger pens and deep straw. This difference could be explained by higher social stress and frustration due to an inability to perform rooting behaviour and to satisfy hunger [15]. Additionally, sows housed in an ESF system scored higher skin lesions than sows housed in gestating stalls due to persistent fighting around the ESF stations [16]. Repeated competition around feeding might, therefore, affect the level of stress in the sow and result in high levels of maternal cortisol, which could decrease PBW [17]. Further, the amount of feed or lack off could potentially increase stress, as Amdi et al. [9] found that restricted fed gilts had higher levels of salivary cortisol than ad libitum fed gilts. The differences in concentration levels of cortisol might therefore be explained by different feeding systems and result in differences in PBW, PBW_CV_, and the occurrence of IUGR piglets between sows of different feed systems.

It, therefore, seems imperative to investigate if different feeding systems can cause different levels of stress to the sow and if the stress is a contributing reason for impaired foetal growth. The aim of this study was, therefore, to investigate the correlation between the overall herd stress level and PBW, IUGR, and PBW_CV_ on selected sows farrowing on the same sampling day. For that, two hypotheses were developed: the level of stress in the sow during gestation, measured by cortisol, can (1) be affected by the feeding system, and (2) affect foetal development, measured by the percentage of IUGR piglets in a litter, PBW, and PBW_CV_ at herd level.

## 2. Materials and Methods

### 2.1. Ethics Statement 

All animals originated from commercial production facilities. No measurements were made that were outside of the standard industry animal husbandry techniques, and the animals were cared for in compliance with local legal standards. The health and welfare of all animals were monitored throughout the sampling days by farm staff, according to the farms’ standard operating protocols and veterinary recommendations. 

### 2.2. Animals and Design

The study was conducted on 12 commercial Danish pig herds with herd populations ranging from 800 to 3050 sows. Three different feeding systems could be observed in the gestation units: Free-access feeding Stall (Stall), Floor feeding (Floor), and Electronic sow feeding (ESF; *n* = 4 herds per system). The herds were selected based on the feeding system in the gestation unit and, secondly, a large herd size in order to record as many litters in each parity over three days as possible. Seven of the herds were selected due to their involvement in other SEGES Danish Pig Research Centre research projects (herd A, C, D, F, I, K, and L), and the remaining herds (B, E, G, H, and J) by contact to a local pig production advisor. Additionally, the herds were selected due to their geographical position, so that farm visits in early mornings and evenings were possible. The herd size and disease status of the herds can be found in Table 1. The study included production data from 8677 liveborn piglets, from a total of 9652 piglets from 452 litters. All sows were crossbred Danish Landrace × Danish Yorkshire (parity 1 to 10 (mean ± sd.; 3.84 ± 1.97)), artificially inseminated with semen from DanBred Duroc boars. Data were collected between mid-September and mid-December 2019. Recordings were made successively over the three days, with the most farrowing in one given week on each herd resulting in the average recording of 40–76% of all farrowing in the weekly farrowing batch of the herds. 

### 2.3. Management Routines

The daily management routines were performed as usual in the individual herds, and recordings were collected early in the morning to prevent disturbing the work routines of the employees. In all herds, sows were housed in a crated system through the nursing period and confined in locked or free-access stalls for four weeks after insemination. The size of the groups in the gestation unit, as well as the number of daily feedings, are listed in Table 1. The herds followed the Danish legislation for stocking density, percentage of slatted floors, and enrichment. Sows in herd G were housed in a deep litter section in the gestation unit. Gilts in herd A were not housed in a Stall system as the sows but in pens with nine gilts in each and fed by liquid feed in a trough. The feed used for gestation diets was formulated to meet or exceed the feed recommendations for gestating sows in all herds [18], and a reduced version of diet formulations can be found in Supplementary Table S1.

### 2.4. Saliva Collection

At least 30 saliva samples were collected from sows in the gestation unit of each herd on the days the production data was collected. Therefore, the saliva samples did not correspond to the sows that farrowed the piglets included in the study. Saliva was collected with the use of a Salivette (Salivette plain, Sarstedt, Leicester, UK), which consisted of a cotton bud that fits inside a centrifuge tube [19]. The sow chewed voluntarily on a cotton bud until it was moisturised, as this method allowed cortisol sampling in an easy and stress-free manner [19,20]. The saliva samples were collected in the morning between 08:00 a.m. and 11:30 a.m., which was either before or at least half an hour after feeding. The sows were selected randomly; however, in herds with ESF, sows seen eating were not selected to avoid contamination of saliva samples. The stocking density in the pen was noted (Table 1), as well as sow ID, parity of the sows, and days from insemination. The saliva samples were centrifuged (CM-6MT; ELMI Ltd., Riga, Latvia) at 1000× *g* for 10 min at room temperature within four hours of collection. Afterwards, the centrifuged saliva samples were transferred to Eppendorf tubes and frozen at −18 °C until later analysis. 

### 2.5. Data on Farrowing Sows

For each sow with a newborn litter, sow ID, date of farrowing, parity, backfat thickness, number of total born, liveborn, stillborn, and mummified piglets were recorded. Backfat thickness from the P2 site, number of days from last weaning to first insemination, length of the previous lactation, and length of gestation were also recorded but not used for this specific study (data not shown). Feed curves and diet formulations were collected at the herds. 

### 2.6. Recordings of Piglets

**Figure 1:**
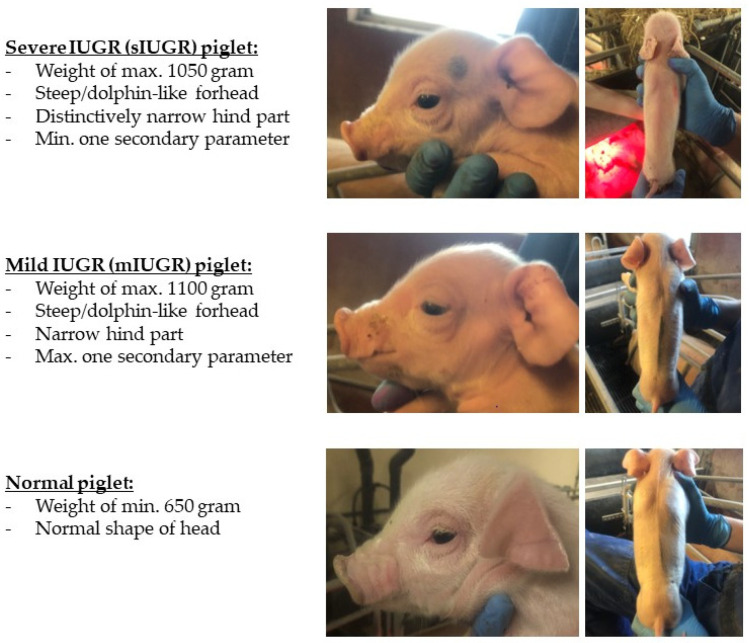
Definition of severe intrauterine growth restriction (sIUGR), mild intrauterine growth restriction (mIUGR), and normal piglets.

Recordings of piglets were carried out as soon as possible after farrowing had ended and before litter equalisation, so they were no older than 24 h at the time of weighing and IUGR scoring (Figure 1). When possible, both live- and stillborn piglets were individually scored as either normal, mild IUGR (mIUGR), or severe IUGR (sIUGR), and the sex of the piglets was recorded. Some dead piglets were removed before registrations were carried out, and in this case, the number of stillborn piglets was only counted by employees, and sex and IUGR score were not noted. Dead piglets were classified as either stillborn or liveborn but dead and, if possible, the cause of death was noted for liveborn but dead piglets. Dead and wet fully formed piglets with the periople still present on the hooves were noted as stillborn. Test of inflation of the lung tissue was not performed. Piglets were noted as liveborn but dead if the above-mentioned criteria of stillborn piglets were not fulfilled. The reason of death was noted as either (1) crushed if visible trauma or subcutaneous edema appeared on any part of the body; (2) euthanised if clear signs of head trauma due to euthanisation was visible; or (3) others if no signs of either (1) or (2) could be detected. 

All liveborn (including liveborn but dead) piglets were individually weighed by placing the piglet in a bucket hanging on a digital weight (5 g weight interval; Ryom Digital Hanging Scale, Hatting, Denmark). To minimise the risk of disease spreading, a new digital weight was used at each herd (accuracy of each weight; ± 25 g deviation). Piglets were scored as either normal, mild IUGR (mIUGR), or severe IUGR (sIUGR). The parameters for sIUGR and mIUGR were based on modified characteristics from Chevaux et al. [21], Hales et al. [5], and Engelsmann et al. [22]. The primary parameters characterizing IUGR piglets were defined as steep/dolphin-like forehead, narrow hind part, and low birth weight (below 1100 g). Secondary parameters were defined as bulging eyes, wrinkles perpendicular to the mouth, spiky hair, and unstable mobility. The sIUGR piglets showed all primary parameters distinctively, had at least one of the secondary parameters, and a weight of no more than 1050 g. Piglets characterised as mIUGR had the primary parameters with a weight of a maximum of 1100 g and no more than one of the secondary parameters. A normal piglet had none of the parameters and weighed more than 650 g. Figure 1 illustrates the distinction between sIUGR, mIUGR, and normal piglets according to the shape of the head and the hind part.

### 2.7. Salivary Cortisol Analysis

Samples with insufficient amounts of saliva (<100 µL) were excluded from the analysis resulting in between 19 and 31 samples analysed for each herd. Saliva cortisol was measured by ELISA (Saliva Lab Trier, daacro GmbH & Co. KG, Trier, Germany), and the inter-assay was 3.35 CV %. Two samples were above the upper limit of quantification of 82.77 nmol/L, which might be due to blood contamination, and were therefore excluded from further analysis.

### 2.8. Data Management and Statistical Analysis

All data management and analysis were performed in RStudio Version 1.2.503 © 2009–2019. The dependent variable in the analysis was salivary cortisol concentration (nmol/L) for individual sows. The study unit was, therefore, the sow. Outliers in the cortisol level data were removed based on two standard deviation criteria, which resulted in the exclusion of 17 observations. Salivary cortisol concentrations were log-transformed to improve the distribution of residuals. The independent variables initially tested for model inclusion were gestation days, feeding system, herd, parity, litter size, and average piglet weight. Parity data as a numeric variable did not follow a linear relation to the outcome and, therefore, was categorised as four groups to improve the fitting: 0–1, 2–3, 4–5, and ≥6. All variables were also checked for confounding and interactions. Model building was based on univariable tests, for which statistical significance was accepted at *p* < 0.05, and 0.05 < *p* < 0.10 was considered a tendency.

All variables which showed a significant effect or a tendency were included in the multivariable models. The variables were then tested for significance as part of mixed-effects models, and ANOVA tests were run between different combinations of variables to assess any differences in fit and explanatory power. 

The following linear mixed-effects model has the final structure used to estimate the effect of parity and gestation days on the saliva cortisol levels:

Y*_i_*= *µ* + *β_j_* + H*_(l)_*+ F*_(k)_* × *α_i_* + *ε_ijkl_* (1)

where Y*_i_* is the response variable (saliva cortisol level); *µ* is the overall mean (intercept); *β_j_* is the fixed effect of gestation days (*j* = (1, 2, …, 113)) explained as a covariate; H*_l_* is the random effect of the herd (*l* = (A, B, …, L)); F*_k_* × *α_i_* is the interaction term between feed system (*k* = (Stall, Floor, ESF)) and parity (*i* = (0–1, 2–3, 4–5, ≥6)); and *ε_ijkl_* is the residual error component, which is assumed to be independent and normally distributed.

The model was run with the help of package lme4 in R [23] (lmer), and the random effect structure was assessed using the Restricted Maximum Likelihood (REML) estimation (RMLE=TRUE). Results were presented as least square means (lsmeans) and their standard errors (SE), as obtained with the help of package lsmeans [24]. Although the difference in degrees of freedom at the different levels of a mixed-effects model did not allow for a clear evaluation of significance through the computation of p-values, a reasonable approximation could be obtained using the package lmerTest, which used Satterthwaite’s degrees of freedom method [25]. These approximated *p*-values will be presented here, along with the regression estimates.

## 3. Results

### 3.1. Salivary Cortisol Concentrations

The average salivary cortisol concentrations across the feed systems at different parities are presented in Table 2, and the results of the linear mixed-effects regression can be found in Supplementary Table S2. To facilitate comprehension, cortisol concentrations are presented as natural values (nmol/L), as the exponential of the logged coefficients and means produced by the model. 

**Figure 2:**
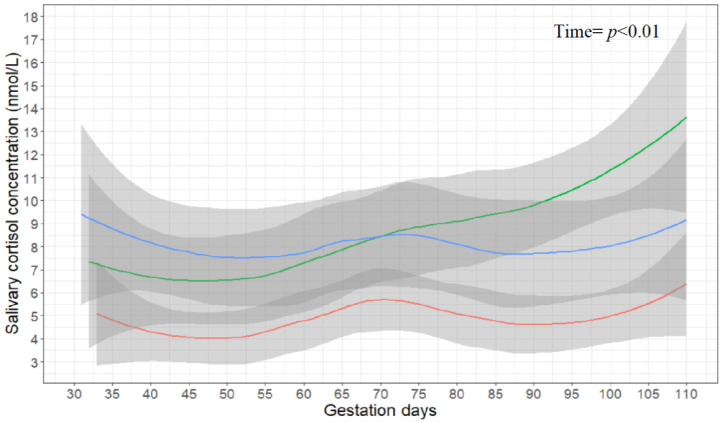
Salivary cortisol concentrations (nmol/L) during gestation in the three feed systems with the 95% confidence interval marked in grey for the free-access feeding stalls (Stall, red), floor feeding (Floor, green), and electronic sow feeding (ESF, blue).

Statistically significant independent effects on salivary cortisol concentrations were observed for gestation days and feeding system. It was estimated that, for each one-day increase in gestation, the sow’s salivary cortisol concentration should increase by 1.00 nmol/L on average (*p* < 0.01). Figure 2 illustrates salivary cortisol concentrations (untransformed observed data) between 30 and 100 days of gestation for individual feeding systems. The plotted values were obtained using a loess smoothening function, with the grey bands corresponding to an approximation of a 95% confidence interval calculated as each smoothed value ± 1.96 × standard errors.

Lower concentrations of salivary cortisol were observed, on average, among sows belonging to the feeding system Stall (4.80 nmol/L) when compared to Floor (7.03 nmol/L) and ESF (7.87 nmol/L), and that difference was statistically significant as an independent effect in the case of ESF (*p* < 0.01). There was no significant independent difference between Floor and ESF or Stall and Floor. A significant interactive effect was also observed between parity and feeding system, with parities 4–5 in ESF herds having a lower increasing effect than other parities in this same system (*p* = 0.02). This could be interpreted as—although sows in herds with an ESF feeding system had, on average, significantly higher concentrations of salivary cortisol than sows in Stall herds—the difference in concentrationwas not as large when Stall sows of any parity were compared specifically with ESF sows in the fourth or fifth parity (Table 2). A similar trend was also observed for ESF sows in parity 0–1 (*p* = 0.08). No interactive effects involving the Floor feeding system or other parity groups were observed.

Variation between herds (random effect) accounted for an additional standard deviation of 1.19 nmol/L to sow-level predictions (Table S3). On that note, the average cortisol concentration in herd G (5.88 nmol/L) was reasonably lower than in F (8.99 nmol/L), J (8.32 nmol/L), and L (8.44 nmol/L), and in the other herds within the ESF system. For a matter of diagnostics, the models were also run without that herd. Omitting herd G increased the mean cortisol level of the ESF system and, consequently, increased the coefficients and significance level for the independent and interactive effects related to the feeding system. It did not, however, alter which predictors were found important in the previous model, nor uncovered new relationships, and the authors considered that the observed values for herd G were valid as it differed from the other ESF herds by being the only one that uses deep straw bedding. Therefore, the final analyses included that herd. Other tendencies or differences between parities within feed systems were not detected.

### 3.2. Production Data

**Figure 3:**
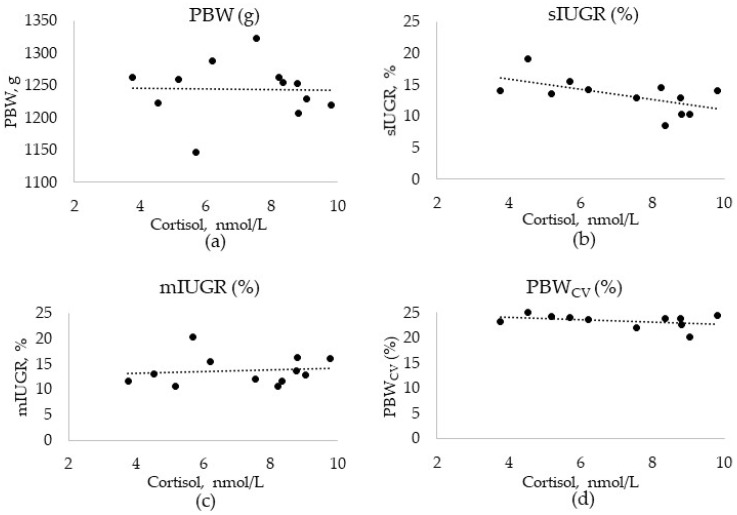
Herd levels of salivary cortisol concentrations (nmol/L) during gestation compared to the herd (**a**) LSmeans of piglet birth weight (PBW), (**b**) percentage of severe IUGR (sIUGR), (**c**) percentage of mild IUGR (mIUGR), and (**d**) within litter variation of PBW (PBW_CV_) for herds within feed systems for sows of 2nd to 9th parity on the sample day.

The average days in gestation when the saliva samples were collected were 67.2 days for ESF, 72.4 days for Floor, and 68.6 days for Stall. The average herd salivary cortisol level and the recorded average herd PBW, sIUGR, mIUGR, and PBW_CV_ from the sows that were in the farrowing unit the day the saliva samples were collected in the gestation unit can be seen in Figure 3. The sows from which the production data was collected were not the same as the ones providing cortisol samples; therefore, no analyses at the sow level were possible. The data were summarised at the herd level to obtain an ecological overview, but the resulting sample size of 12 herds was too small for any kind of inference. 

## 4. Discussion

The aim of this study was to measure stress levels on herd level measured by cortisol in sows housed with different feed systems. Salivary sampling provided a non-invasive, low-impact sampling of cortisol, and was considered just as representative as blood cortisol for the short-term levels of stress in the animal [19,20]. 

In the present study, no connections between herd cortisol levels and the results of PBW and the occurrence of IUGR piglets (both severe and mild) were evident. However, it must be stressed that this was an ecological study as the cortisol levels were not from the same sows that the piglet information came from, but it was the overall herd performance compared to the overall herd cortisol level during gestation. Therefore, several factors were not taken into account, such as genetics, individual variation, exercise, and stocking density that could all influence cortisol levels. The observed increase of cortisol throughout gestation was most likely a biological response as cortisol peaked around parturition [26] in sows and dropped again throughout the first few weeks of lactation [27].

It is not known what level of severity of cortisol exposure is required to have an effect on PBW. In previous studies, the effect of prenatal stress was investigated by manipulating maternal cortisol concentrations with oral administration of hydrocortisone acetate (HCA), injection of adrenocorticotropic hormones (ACTH) [28], or by stressful events such as rough handling or frequent social mixing [29,30]. Neither rough handling, social mixing, or ACTH injection affected the PBW, whereas sows subjected to HCA had lighter piglets [17,28,30,31]. Hydrocortisone acetate was metabolised directly to cortisol in the body [32], whereas ACTH stimulated the adrenal cortex to release cortisol [7]. Rough handling and social-mixing-imposed stress on the sows, which could be detected by increased cortisol concentrations in saliva or blood, though not to the same level as administered ACTH did [30,31]. The sows in the study by Kranendonk et al. [17] were given HCA for 30 days in early, mid, or late gestation. The offspring of the sows, which received the treatment in early and late gestation, had the lowest PBW whilst the offspring of the sows treated in mid-gestation were intermediate in relation to the control group, which had the highest PBW [17]. The contradictory results might be explained by the timeframe in gestation that the treatment was given and the severity of the prenatal stress imposed. The levels of cortisol of the stressors were much higher in the HCA study than what other studies reported ([9,20]. The HCA treatment elevated the salivary cortisol levels to 23.3–29.9 ng/mL [17], equal to 64.3–82.5 nmol/L, which were very high compared to the level reported by Cook et al. [20] of sows stressed by handling and transport (11.2 nmol/L) and the levels found in the current study. The HCA was also administered every day for 30 days, whereas the sows were exposed to the other stressors less frequently. At last, HCA had the lowest impact in mid-gestation [17], which was the period that one of the studies with ACTH treatment and rough handling was conducted [30]. When ACTH was administered to sows in early pregnancy, it tended (*p* = 0.09) to affect the PBW [28]. This indicated the importance of the time of stressors. Therefore, it was likely that HCA created high levels of cortisol continuously for a period long enough to damage the foetal growth, which was not the case for the other stressor studied. This could indicate that maternal stress must be quite severe or perhaps long-term to affect foetal growth, even though it was unclear how stressed the sow must be in a commercial setting before that happens.

Feeding systems did affect saliva cortisol concentrations, but the effect on productive performance seemed more complex. Maternal saliva cortisol concentrations were higher in the morning (9:30 a.m.) prior to feeding than later in the day (12:30 and 15:30 p.m.) and were also affected by diet restrictions and body condition [9]. Amdi et al. [9] found that gilts fed a restricted diet (1.8 kg/day) from day 25 of gestation had higher saliva cortisol levels (8.50 nmol/L) than gilts fed a high feeding level (3.5 kg/day, 5.00 nmol/L) in the same period. In addition, thin gilts (14.8 mm backfat) also tended to have higher saliva cortisol levels, 7.34 nmol/L, than fat gilts (20.2 mm) at 6.5 nmol/L [9]. Additionally, feeding frequency (one or two feedings per day) was found to not affect saliva cortisol concentrations of individually stalled gilts in a study by Holt et al. [14]. The average level of saliva cortisol in this study was in line with earlier studies on gestating sows [9,20]. Even though the cortisol levels in sows under the Floor and ESF systems (7.03 and 6.36 nmol/L, respectively) were higher than those in Stall (4.01 nmol/L), these levels were still close to earlier reports of individually stalled gestating sows subject to no stressors (5.8 nmol/L [20]), and also below the levels reported for gestating sows subjected to common stressors such as handling and transport (11.2 nmol/L [20]). This was in agreement with Anil et al. [13], who found that at any given point in gestation, salivary cortisol concentrations were higher in sows housed in the ESF system than for sows housed in individual stalls, but the differences in cortisol concentrations between systems did not seem to affect PBW [13]. This could indicate that, in general, the level of stress in the sows was not high enough to affect the development of the piglets even though it differed between systems. One of the farms (G) had straw in the ESF system and, on average, lower levels of salivary cortisol than the other farms with the ESF system. This indicated that several factors, for example, housing and management in the different feed systems, should be considered together. In addition, cortisol production showed large inter-individual variation, which had a considerable genetic basis [33], and this should, therefore, also be considered. Additionally, stocking density could influence welfare indicators, but with no difference in cortisol between groups in growing pigs [34], suggesting multiple factors should be included in order to assess the full physiological response of the feeding systems. In addition, it could be discussed if the absolute values were the most accurate way of assessing cortisol levels or if differences from, for example, a herd baseline level would be more accurate due to individual variation. We had previously reported salivary cortisol values in differences from a baseline value [27], and perhaps herd cortisol levels need to be assessed continuously throughout a longer period to establish a herd baseline.

It was shown that dynamic group management in ESF did not affect maternal saliva cortisol concentrations when compared to static groups [12], but increasing floor space allowance (from 1.4 m^2^/sow to 3.0 m^2^/sow) decreased aggression and plasma cortisol levels around mixing [35,36]. An enriched environment (deep straw, 3.4 m^2^/sow) lowered the maternal saliva cortisol concentrations in both early and late gestation compared to sows in conventional housing (slatted floor, 2.4 m^2^/sow) [15,37,38]. In agreement, contradicting effects on PBW were reported in three different studies, which tested the effect of enriched or conventional housing [15,37,38]. One study reported lower PBW from conventionally housed sows [37], contradicting another study by the same authors, where they found no effect of housing [15]. Even though enriched or conventional gestational housing had no effect on mean PBW (~1.5 kg), a higher percentage of lower birth weight piglets (<1.2 kg) was reported from sows of conventional housing [15,38] and a higher percentage of heavy birth weight piglets (>2.0 kg) was seen from the enriched facility [38]. The sows in enriched housing had salivary cortisol concentrations of approximately 3.45–4.14 nmol/L, whereas the concentrations were approximately 6.90–9.66 nmol/L for sows in conventional housing (converted values from ng/mL from Quesnel et al. [38]). This suggested that the maturity of the piglets at birth was positively affected by lower salivary cortisol during gestation. As the cortisol levels in the current study were not measured from the same sows the piglets were born from, the levels of cortisol were only indicative of the general level of stress in the different systems, with no direct connection to the observed litters. 

## 5. Conclusions

Feeding systems during gestation seemed to have an effect on the stress level of sows, as reflected by their level of salivary cortisol. However, it was not possible to confirm that the differences in stress between systems had an impact on PBW or on the occurrence of sIUGR piglets at herd or litter level due to limitations in the study design. Further studies are warranted to investigate this relationship as this could potentially improve welfare and productivity in pig production systems in the future. 

## Figures and Tables

**Table 1:** Number of feedings per day and sows per pen in the gestation unit for the individual herds (A–L) with free-access feeding stalls (Stall), floor feeding (Floor), and electronic sow feeding (ESF).

	Stall				Floor				ESF			
Herd	A	C	D	I	B	E	H	K	F	G	J	L
Herd size, no. of sows	1900	1250	1050	1150	3050	2500	1250	2000	800	1200	1400	1700
Health status ^1^	Myc + Ap12	Myc + Ap6 + Ap12	Myc	Myc + Ap6 + Ap12	Myc + PRRS1 + PRRS2	SPF	Myc + Ap12	Unknown	Myc + PRRS2	Myc + Ap12	Myc + Ap2 + PRRS1 + PRRS2	Unknown
Feedings/day, no.	1–2	1–2	2	2	1	2	1	1	-	-	-	-
Sows/pen in the gestation unit, no.	60–65	50	46	25–40	30–56	50–60	16 or 40–45 ^2^	18	45–60	60–65	48–64	75

^1^ Health status according to the Danish SPF declaration system: SPF = Specific pathogen free; Myc = *Mycoplasma hyopneumoniae*; Ap2 *= Actinobacillus pleuropneumoniae* serotype 2; Ap6 *= Actinobacillus pleuropneumoniae* serotype 6; Ap12 *= Actinobacillus pleuropneumoniae* serotype 12; PRRS1 = Porcine Reproductive and Respiratory Syndrome (EU strain); PRRS2 = Porcine Reproductive and Respiratory Syndrome (US strain); Unknown = no health declaration. ^2^ Two different pen sizes were present in this herd.

**Table 2:** Salivary cortisol concentrations (nmol/L) of sows at different parities in different feed systems with free-access feeding stalls (Stall), floor feeding (Floor), and electronic sow feeding (ESF) presented as least square means (lsmeans) and standard errors (SE). The parity reflects the current parity at gestation, so parity 0 equals gilts pregnant with their first litter and so on.

Feed System	Parity 0–1		Parity 2–3		Parity 4–5		Parity ≥ 6	
	Lsmean	SE	Lsmean	SE	Lsmean	SE	Lsmean	SE
Stall (nmol/L)	4.97	1.15	3.60	1.13	4.21	1.15	3.03	1.30
Floor (nmol/L)	7.45	1.14	7.36	1.15	6.71	1.18	5.77	1.44
ESF (nmol/L)	7.61	1.14	6.09	1.14	5.03	1.17	8.62	1.25

## Data Availability

Data are available upon request.

## Supplementary Materials

The following are available online at https://www.mdpi.com/article/10.3390/ani11041074/s1. Table S1—Reduced feed formulation of the gestation diet in the individual herds in different feed systems: free-access feeding stalls (Stall), floor feeding (Floor), and electronic sow feeding (ESF) for the twelve farms included in the study (A–L). Supplementary Table S2—Linear regression coefficients and lsmeans. Supplementary Table S3—Mean cortisol level by herd divided by parity group and feed system.
