# Supplementary material for: The Effect of Different Feeding Systems on Salivary Cortisol Levels during Gestation in Sows on Herd Level
Source: Animals (Basel). 2021 Apr 9;11(4):1074. doi: 10.3390/ani11041074 (PMC8070664; doi:10.3390/ani11041074)
Supplement: Supplementary file 1 [file animals-11-01074-s001.pdf]

The Effect of Different Feeding Systems on Salivary Cortisol Levels during Gestation in Sows on Herd Level

Ida Bahnsen [1,†], Kristina V. Riddersholm [1,†], Leonardo V. de Knegt [1], Thomas S. Bruun [2] and Charlotte Amdi [1,*]

[1] Department of Veterinary and Animal Sciences, Faculty of Health and Medical Sciences, University of Copenhagen, Grønnegårdsvej 2, 1870 Frederiksberg, Denmark; idabahnsen@yahoo.dk (I.B.); riddersholm95@hotmail.com (K.V.R.); lvdk@sund.ku.dk (L.V.d.K.)

[2] SEGES Danish Pig Research Centre, Axeltorv 3, 1609 Copenhagen, Denmark; thsb@seges.dk

* Correspondence: ca@sund.ku.dk

† Joint first authors

Supplementary Table S1. Reduced feed formulation of gestation diet in the individual herds in different feed systems; free-access feeding stalls (Stall), floor feeding (Floor) and electronic sow feeding (ESF) for the twelve farms included in the study (A-L).

	**Stall**				**Floor**				**ESF**			
**Herd**	**A**	**C**	**D**	**I**	**B**	**E**	**H**	**K**	F	G	J	L
MJ NE/kg feed	8.9	9.3	9.4	9.1	9.5	9.3	9.0	9.7	9.6	9.5	9.0	9.3
Crude protein (g SID/kg)	92	97	98	94	100	96	93	119	105	107	98	110
Lysine (g SID/kg)	3.88	5.38	5.15	5.13	5.20	5.05	4.61	4.94	5.61	6.24	5.10	5.85
Methionine (g SID/kg)	1.70	1.72	1.55	1.50	1.77	2.22	1.55	2.13	1.79	1.98	1.73	1.79
Methionine + Cysteine (g SID/kg)	3.58	3.65	3.46	3.23	3.84	4.14	3.40	4.10	3.76	4.02	3.69	4.02
Threonine (g SID/kg)	3.11	3.38	3.71	3.69	3.21	3.33	3.15	3.22	3.47	4.06	3.53	4.25

Supplementary Table S2. Linear regression coefficients and LSmeans.

Random effect	SD				
H2	1.185305				
Residual	1.798944				
**Fixed Effects**	**Estimate**	**SE**	**df**	***t***	***p***
Intercept	2.31	1.31	175	3.08	0.002
Gestation days	1.00	1.00	275	2.86	0.005 *
Parity					
0–1	1.65	1.30	270	1.87	0.062
2–3	1.19	1.30	273	0.67	0.501
4–5	1.40	1.31	272	1.23	0.220
Feed system					
Floor	1.91	1.56	206	1.46	0.146
ESF	2.85	1.41	151	3.06	0.003 *
Interactions					
Parity 0–1 * Floor	0.78	1.57	274	-0.54	0.587
Parity 2–3 * Floor	1.07	1.57	275	0.15	0.884
Parity 4–5 * Floor	0.83	1.59	274	-0.40	0.693
Parity 0–1 * ESF	0.54	1.43	271	-1.76	0.080
Parity 2–3 * ESF	0.59	1.42	272	-1.50	0.135
Parity 4–5 * ESF	0.42	1.44	272	-2.37	0.018 *

Feed system	Parity	LSmeans	SE	df	Conf. Limits	n
Stall	0-1	4.97	1.15	27	3.04–8.13	29
	2-3	3.6	1.13	17	2.27–5.72	42
	4-5	4.21	1.15	30	2.57–6.92	26
	≥ 6	3.03	1.3	163	1.32–6.93	6
Floor	0-1	7.45	1.14	20	4.65–11.91	38
	2-3	7.36	1.15	24	4.52–11.97	31
	4-5	6.71	1.18	44	3.88–11.62	19
	≥ 6	5.77	1.44	223	1.82–18.34	3
ESF	0-1	7.61	1.14	24	4.72–12.27	32
	2-3	6.09	1.14	22	3.79–9.77	34
	4-5	5.03	1.17	42	2.96–8.55	20
	≥ 6	8.62	1.25	128	4.17–17.84	8

Supplementary Table S3. Mean cortisol level by herd, divided by parity group and feed system.

Feed system	Parity	Herd	Cortisol level		*n* animals
			mean	sd	
ESF	0–1	F	8.97	5.41	9
		G	6.83	4.36	9
		J	10.74	3.45	5
		L	9.19	4.82	9
	2–3	F	9.30	5.70	9
		G	6.15	6.99	9
		J	8.21	3.89	9
		L	6.87	6.96	7
	4–5	F	3.17	0.02	2
		G	4.72	2.05	6
		J	4.56	1.62	5
		L	8.75	6.48	7
	≥ 6	F	12.04	3.69	3
		G	2.01	-	1
		J	10.86	5.90	3
		L	10.41	-	1
Stall	0–1	A	4.54	2.92	3
		C	7.09	1.75	5
		D	3.69	2.16	11
		I	7.78	4.25	10
	2–3	A	5.56	3.81	9
		C	3.42	2.11	13
		D	3.00	1.61	10
		I	5.51	3.26	10
	4–5	A	4.36	1.50	8
		C	5.29	4.96	6
		D	4.44	2.26	6
		I	6.17	4.10	6
	≥ 6	A	2.61	-	1
		D	2.94	1.75	4
		I	2.88	-	1
Floor	0-1	B	12.42	7.72	5
		E	7.93	5.95	11
		H	9.75	3.58	11
		K	8.06	4.53	11
	2–3	B	7.32	5.05	11
		E	9.85	6.49	5
		H	7.76	4.21	4
		K	10.00	6.02	11
	4–5	B	5.23	3.89	3
		E	6.94	4.60	6
		H	10.91	7.22	9
		K	10.47	-	1
	≥ 6	E	5.48	1.77	3
